# Autoimmune Hepatitis with Distal Renal Tubular Acidosis and Small Bowel Partial Malrotation

**DOI:** 10.5005/jp-journals-10018-1145

**Published:** 2016-07-09

**Authors:** Tejas Kanaiyalal Modi, Hardik Parikh, Abhishek Sadalge, Amit Gupte, Pratin Bhatt, Akash Shukla

**Affiliations:** 1 Department of Gastroenterology, Seth GS Medical College, KEM Hospital, Mumbai, Maharashtra, India

**Keywords:** Autoimmune hepatitis, Malrotation, Renal tubular acidosis.

## Abstract

Renal tubular acidosis (RTA) is not uncommon in patient with chronic autoimmune hepatitis (AIH), but usually remains latent. Here, we report a case of renal tubular acidosis RTA who presented with AIH. She was also diagnosed to have partial bowel malrotation. A 9-year-old girl, a case of distal RTA, presented with jaundice, abdominal distension and altered sensorium. She was diagnosed to be AIH, which was successfully treated with steroids and azathioprine. Coexistent midgut partial malrotation with volvulus was diagnosed during the treatment. She was treated successfully with anti-tuberculous treatment for cervical lymphadenitis. Autoimmune hepatitis should not be ruled out in each case of RTA presenting with jaundice.

**How to cite this article:**

Modi TK, Parikh H, Sadalge A, Gupte A, Bhatt P, Shukla A. Autoimmune Hepatitis with Distal Renal Tubular Acidosis and Small Bowel Partial Malrotation. Euroasian J Hepato-Gastroenterol 2015;5(2):107-109.

## INTRODUCTION

Chronic autoimmune liver disease and renal tubular acidosis (RTA) are frequently associated, possibly related to their common autoimmune pathogenesis.^1^ In such cases of autoimmune hepatitis (AIH), diagnosis of RTA is usually done by inability of the kidney to handle acid load.^2^ But, here we present a case of renal tubular acidosis (RTA) which was diagnosed at the age of 4 years on regular treatment, now presenting with AIH which was successfully treated with steroids and azathioprine. She was also diagnosed to have tuberculous lymphadenitis and partial malrotation of midgut.

## CASE REPORT

**Fig. 1:**
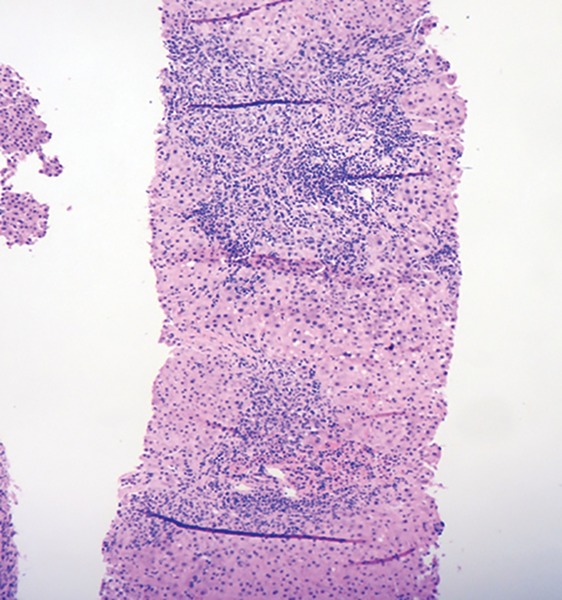
Liver biopsy showing dense portal and interface inflammation. (HE, 100x)

**Fig. 2:**
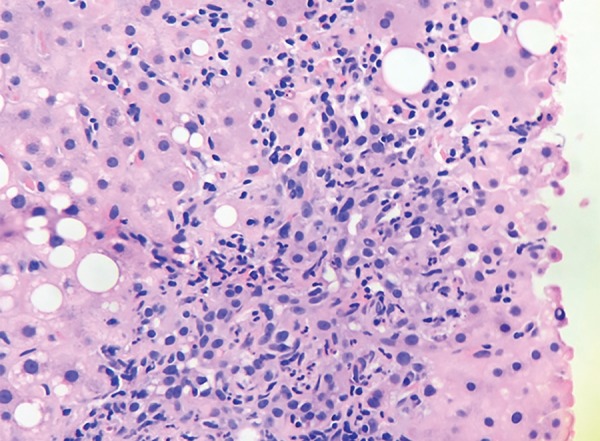
Closer view shows portal and interface inflammation with predominantly lymphocytes and plasma cells. Bile ductular proliferation is also noted. Hepatocytes show minimal steatosis (<10%). (HE, 400x)

**Fig. 3:**
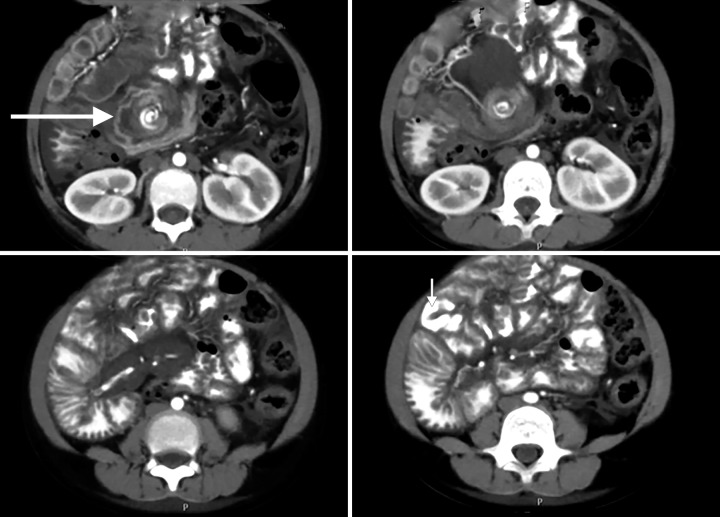
Contrast enhanced computed tomography shows duodenojejunal flexure on right side of spine with jejunal loops (filled with iodinated contrast predominantly on right side (small arrow). There is evidence of twisting of mesenteric vessels surrounding small bowel (whirlpool sign); midgut volvulus (large arrow)

**Fig. 4 :**
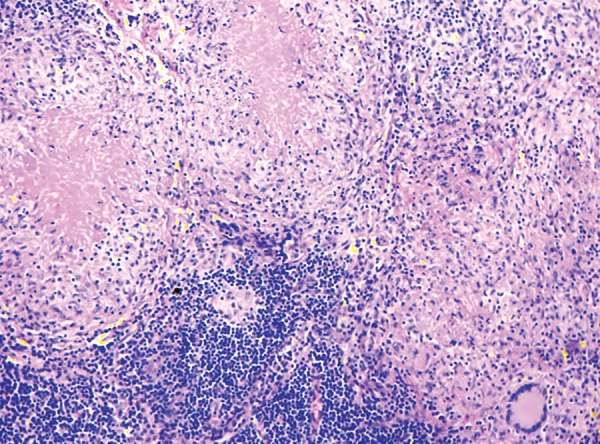
Lymph node showing confluent epithelioid cell granulomas with Langhan’s giant cells and caseous necrosis (HE, 100×)

A 9-year-old girl presented with past history of acute onset abdominal pain, bilious vomiting and abdominal distension at 4 years of age. Investigations (X-ray abdomen and ultrasound abdomen) showed multiple air fluid levels with dilated fluid filled bowel loops. Her height was 85 cm and weight was 10 kg at 4 years (both were below 3rd percentile). Her blood investigation revealed persistent hypokalemia (potassium 2.5 mg/dl). She was treated conservatively. Six months later she again developed similar episode along with hypokalemia. She also had normal anion gap acidosis along with hyperchloremia (venous blood pH 7.28, serum chloride 113 mg/dl). She was also found to have hypercalciuria (urine calcium 22.88) (urinary calcium/creatinine ratio 1.4) with high urinary pH (8.0). On reviewing history further, she was born of non-consanguineous marriage, had birth weight of 2.5 kg and normal milestones till 1 year of age. Later on, mother had noticed low height and low weight gain compared to other child. She also noticed polyuria, polydipsia and nocturia since age of 3 years. She was diagnosed as a case of distal RTA and treated with Shohl’s solution (bicarbonate) and potassium chloride. Her height was increased and she gained weight up to 19 kg at 9 years. At this age, she presented with the history of fever, malaise, and fatigue with jaundice without any cholestasis. Few days later she developed ascites and pedal edema. Laboratory investigations revealed bilirubin of 4.9 mg/dl (direct 3.6 mg/dl), aspartate aminotransferase (AST); 739 IU/ml, alanine aminotransferase (ALT); 489 IU/ml, alkaline phosphatase (ALP); 390 IU/ml (UNL -117), gama glutamyl transpeptidase (GGT); 38 (normal), total protein/albumin (6.6 gm/2.3 gm), international naturalized ratio (INR); 2.5. Viral markers such as IgM type hepatitis A virus (HAV), IgM type hepatitis E virus (HEV), Hepatitis B surface antigen (HBsAg), IgM type anti-HBc, and anti-hepatitis C virus were negative. Ultrasound with Doppler investigations showed coarse echo texture with presence of ascites with normal hepatic veins, inferior vena cava and dilated portal vein. Anti-nuclear antibody (ANA) was positive with a titer of 1:320, Anti dsDNA was positive with 1:80 (4+) titers, anti LKM-1 positive with 1:40, IgG was 2500 mg/dl (upper limit of normal: up to 1100 mg/dl), anti-smooth muscle antibody was negative. Investigations for Wilson’s disease was negative (serum ceruloplasmin was 19.8 mg/dl, 24-hour urinary copper was 34 ug (normal < 40 μg), and KF rings absent). Ascites fluid had low protein (0.27 gm) with high serum ascites albumin gradient (SAAG;1.48). She also developed one episode of hepatic encephalopathy and spontaneous bacterial peritonitis during 20 days of hospitalization which was successfully treated with antibiotics. She underwent transjugular liver biopsy and that was compatible with AIH (lobular hepatitis, rosette formation and interphase hepatitis) with early cirrhosis (Figs 1 and 2). Treatment was started with prednisolone and azathioprine. After starting this treatment, she started complaining bilious vomiting and diffuse abdominal pain. She underwent computed tomography scan of the abdomen (Fig. 3) which showed partial malrotation of small bowel with volvulus with proximal dilatation of duodenum with pretracheal, perivascular, mesenteric and peripancreatic lymph nodes. She also had palpable cervical lymph node (≈1.5 cm), excision biopsy of which was suggestive of epithelioid granuloma with caseation (Fig. 4). She was given anti-tubercular drugs with close monitoring of liver function test.

After 3 weeks, liver function test (LFT) was significantly improved with bilirubin 1.2 mg/dl, AST 68IU/l, ALT 75IU/l, and PT/INR 1.5. Serum albumin was 2.8 gm/dl. Pedal edema and ascites decreased and disappeared over a period of 2 months. Three months later bilirubin was 1.3 mg/dl, AST; 55IU/l, ALT; 82 IU/l, ALP; 82, total protein/albumin; 6.2/3.7 gm/dl, PT/INR; 1.0. Her weight increased to 25 kg and height raised to 120 cm over a period of 12 months. Final diagnosis was RTA with AIH and partial small bowel malrotation.

## DISCUSSION

Distal RTA or type 1 RTA is the classical form of RTA characterized by a failure of acid secretion by the alpha intercalated cells of the cortical collecting duct leading to inability to acidify the urine which may be hereditary or may be triggered by an autoimmune disorder.^3^ Distal RTA is commonly associated with autoimmune diseases like Sjogren’s syndrome, systemic lupus erythematous, rheumatoid arthritis, hypergammaglobulinemia and autoimmune liver disease. Previous study found coexistence of RTA in 60% of the patients with primary biliary cirrhosis and in 30% with AIH.^1,2^ In such cases RTA is frequently latent.^4^

Pediatric AIH is of two types. Type 1 is smooth muscle antibody (SMA) and/or antinuclear antibody (ANA) positive, and type 2 is positive for antibodies to liver-kidney microsome type 1 (anti LKM1) with 80% female predominance in both.^5^ Type 2 AIH usually presents more acutely and possible in association with autoimmune disorders, family history of autoimmunity, and IgA deficiency. As in our case, a young girl had two features which possibly suggest type 2 AIH; (1) acute onset of symptoms and (2) prior history of RTA possibly of autoimmune origin. Treatment with steroids and azathioprine has response in 80% of cases, however, relapses are also common. She was treated with prednisolone (2 mg/kg) and azathioprine (1 mg/kg) and her symptoms improved over 2 months. Coexistence of malrotation of gut and autoimmune disorders had been described in certain genetic syndromes.^6,7^ To the best of our knowledge nonsyndromic association of malrotation with RTA with autoimmune diseases has not been described previously.

## References

[1] Golding PL (1975). Renal tubular acidosis in chronic liver disease.. Postgrad Med J.

[2] Golding PL, Mason AS (1971). Renal tubular acidosis and autoimmune liver disease.. Gut.

[3] Karet FE (2011). Disorders of water and acid-base homeostasis.. Nephron Physiol.

[4] Toblli JE, Findor J, Sorda J, Bruch Igartua E, Hasenclever K, Collado HD (1993). Latent distal renal tubular acidosis (dRTA) in primary biliary cirrhosis (PBC) and chronic autoimmune hepatitis (CAH).. Acta Gastroenterol Latinoam.

[5] Mieli-Vergani G, Heller S, Jara P (2009). Autoimmune hepatitis.. J Pediatr Gastroenterol Nutr.

[6] Bassett AS, Chow EW, Husted J, Weksberg R, Caluseriu O, Webb GD, Gatzoulis MA (2005). Clinical features of 78 adults with 22q11 Deletion Syndrome.. Am J Med Genet A.

[7] Hastings R, Cobben JM, Gillessen-Kaesbach G, Goodship J, Hove H, Kjaergaard S, Kemp H, Kingston H, Lunt P, Mansour S (2011). Bohring-Opitz (Oberklaid-Danks) syndrome: clinical study, review of the literature, and discussion of possible pathogenesis.. Eur J Hum Genet.

